# Omecamtiv mecarbil treatment improves post-resuscitation cardiac function and neurological outcome in a rat model

**DOI:** 10.1371/journal.pone.0264165

**Published:** 2022-02-17

**Authors:** Shih-Ni Wu, Min-Shan Tsai, Chien-Hua Huang, Wen-Jone Chen

**Affiliations:** 1 Department of Emergency Medicine, National Taiwan University Medical College and Hospital, Taipei, Taiwan; 2 Division of Cardiology, Department of Internal Medicine, National Taiwan University Medical College and Hospital, Taipei, Taiwan; Scuola Superiore Sant’Anna, ITALY

## Abstract

**Background:**

Myocardial dysfunction is a major cause of poor outcomes in the post-cardiac arrest period. Omecamtiv mecarbil (OM) is a selective small molecule activator of cardiac myosin that prolongs myocardial systole and increases stroke volume without apparent effects on myocardial oxygen demand. OM administration is safe and improves cardiac function in patients with acute heart failure. Whether OM improves post-resuscitation myocardial dysfunction remains unclear. This study investigated the effect of OM treatment on post-resuscitation myocardial dysfunction and outcomes.

**Methods and results:**

Adult male rats were resuscitated after 9.5 min of asphyxia-induced cardiac arrest. OM and normal saline was continuously intravenously infused after return of spontaneous circulation (ROSC) at 0.25 mg/kg/h for 4 h in the experimental group and control group, respectively (n = 20 in each group). Hemodynamic parameters were measured hourly and monitored for 4 h after cardiac arrest. Recovery of neurological function was evaluated by neurological functioning scores (0–12; favorable: 11–12) for rats 72 h after cardiac arrest. OM treatment prolonged left ventricular ejection time and improved post-resuscitation cardiac output. Post-resuscitation heart rate and left ventricular systolic function (dp/dt_40_) were not different between groups. Kaplan-Meier analysis showed non-statistically higher 72-h survival in the OM group (72.2% [13/18] and 58.8% [10/17], p = 0.386). The OM group had a higher chance of having favorable neurological outcomes in surviving rats 72 h after cardiac arrest (84.6% [11/13] vs. 40% [4/10], p = 0.026). The percentage of damaged neurons was lower in the OM group in a histology study at 72 h after cardiac arrest (55.5±2.3% vs. 76.2±10.2%, p = 0.004).

**Conclusions:**

OM treatment improved post-resuscitation myocardial dysfunction and neurological outcome in an animal model. These findings support further pre-clinical studies to improve outcomes in post-cardiac arrest care.

## Introduction

Although cardiopulmonary resuscitation (CPR) has been developed for more than 50 years, sudden cardiac arrest still carries a high mortality rate. The survival-to-discharge rate is less than 20%, even though the return of spontaneous circulation (ROSC) rate is as high as 40–60% [1, 2]. Cardiac arrest survivors experience persistent global ischemia-reperfusion injury for up to 72 h or longer during the post-cardiac arrest period. This injury leads to post-cardiac arrest syndrome, which is a complex combination of pathophysiological processes comprising post-cardiac arrest cerebral injury and myocardial dysfunction and contributes to multiple organ failure and mortality [3, 4]. The low cardiac output caused by myocardial dysfunction exacerbates organ dysfunction and degrades long-term neurological outcomes in cardiac arrest survivors [3, 5–7]. Laurent et al. reported that resuscitated patients with a persistent low cardiac index at 24 h were more likely to experience multi-organ failure and early death [3]. Post-cardiac arrest myocardial dysfunction is defined as global dysfunction due to myocardial stunning, which is potentially reversible and treatable [3, 4, 8]. Improving post-cardiac arrest myocardial dysfunction may ameliorate hemodynamic instability and survival outcomes.

Omecamtiv mecarbil (OM) is a selective small molecule cardiac myosin activator that specifically binds to cardiac myosin without affecting other types of myosin in smooth or skeletal muscle [9]. In the cardiomyocyte contractile cycle, myosin head activation occurs by ATP hydrolysis to ADP and Pi and enables cross-bridge formation between myosin heads and active sites on actin. Afterwards, Pi is released from myosin with a transition from the weakly actin-bound state to a strongly actin-bound state. This transition is a decisive and rate-determining step during the entire actin-myosin ATPase cycle. The actin-myosin complex dissociates when an ATP molecule binds myosin heads, which liberates ADP and releases actin filaments from myosin [10, 11]. OM augments the speed of ATP hydrolysis with consequent Pi release, thus accelerating the transition rate from a weakly bound to a strongly bound actin-myosin complex. OM also increases the number of active cross-bridge formations during systole. The effects of OM on cardiac function are achieved by prolonging systolic ejection time. This results in increased cardiac contractility and stroke volume without changes in the heart rate or apparent effects on myocardial oxygen demand [10–13].

OM administration is safe and improves cardiac function in patients with acute heart failure [11, 12]. However, whether OM improves post-cardiac arrest myocardial dysfunction remains unclear. In the current study, we hypothesized that OM treatment can improve post-resuscitation cardiac function and further survival and neurological outcomes.

## Methods

The study was approved by the Institutional Animal Care and Use Committee (IACUC) of the National Taiwan University College of Medicine and Public Health and complied with guidelines in the *Guide for the Care and Use of Laboratory Animals* published by the US National Institutes of Health. The date ranges of the experiment was between Jun. 2nd 2017 and Jan. 9th 2018. Research staffs received training in the care and use of laboratory animals before the study. All rats were housed in a rodent facility with a 12 h-light/ 12-h dark cycle. Food and water were provided ad libitum for all animals prior to the experiment.

### Animal preparation and hemodynamic monitoring

Male Wistar rats (12 weeks old) were anesthetized with pentobarbital (30 mg/kg) delivered by intraperitoneal injection to minimize the possible suffering and distress for the whole procedures during the procedures and prepared as previously described [14, 15]. If there was any discomfort or agitation, additional anesthetics were given for the animal. The animals were endotracheally intubated through the mouth with a PE 200 catheter (Angiocath, Becton Dickinson) and mechanically ventilated (Flexivent EC-VF-2, Scireq Scientific Respiratory Equipment Inc.). Ventilator settings include a tidal volume of 0.8 mL/100 g, a respiratory rate of 90 breaths per minute, and a FiO_2_ of 1.0. Arterial blood pressure was measured with a saline-filled PE-50 tube inserted through the right femoral artery. To measure left ventricular (LV) pressure, another saline-filled PE-50 tube was inserted through the right carotid artery and advanced to the left ventricle, as confirmed by echocardiography and left ventricular pressure waveforms. Fluids and drugs were administered through the right jugular vein route. A PC-based data acquisition system (ADI Instruments, Inc.) was used to record hemodynamic parameters and needle-probe ECG monitoring data. Hemodynamic parameters were measured every hour and monitored before cardiac arrest to the 4^th^ hour after ROSC. LV ejection time was defined as the duration of blood flow across the aortic valve. LV systolic function was represented by dp/dt at 40 mmHg of LV pressure (dp/dt_40_). The thermocatheter for detecting blood temperature was placed through the left femoral artery and advanced to the thoracic aorta by the fixed length method. Cardiac output was measured using the thermodilution method, which was then analyzed with a Cardio-Max II system (Columbus Instrument, Columbus, OH, USA).

### Asphyxia-induced cardiac arrest animal model and study design

**Fig 1:**
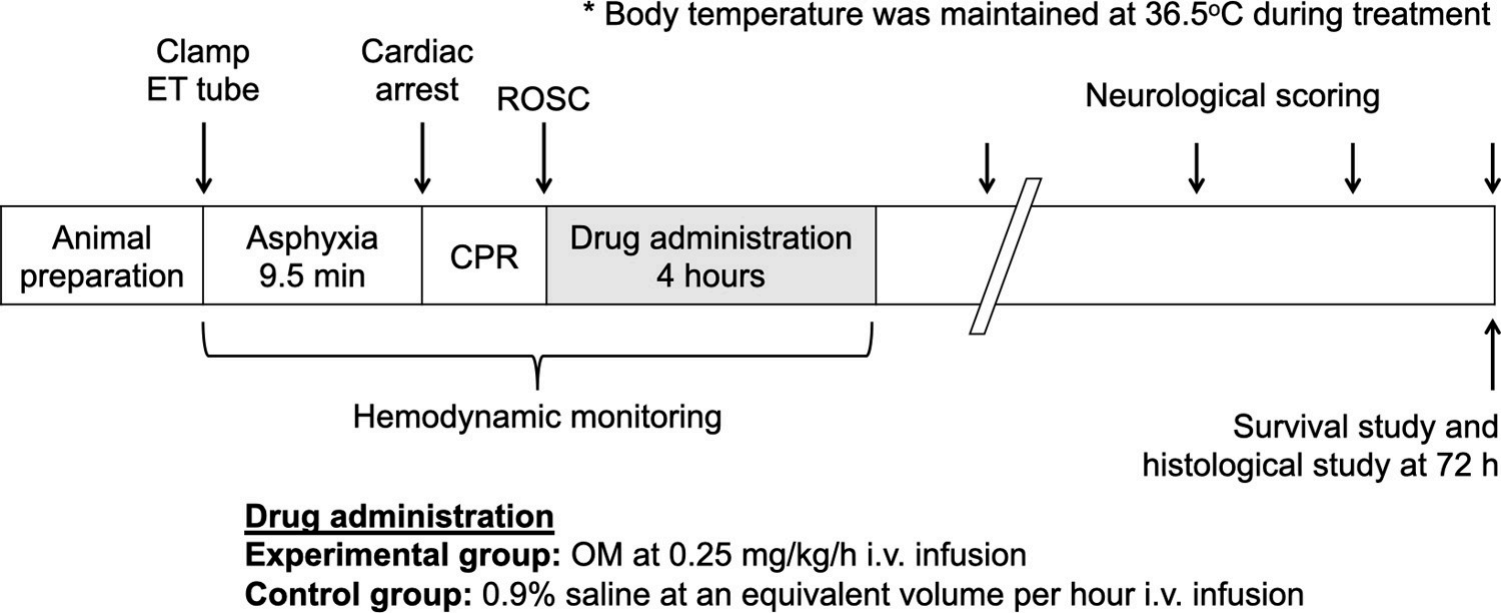
Study design and protocol for inducing cardiac arrest, resuscitation, drug administration, and monitoring.

Cardiac arrest was induced by asphyxia by turning off the ventilator and clamping the endotracheal tube. Bradycardia and hypotension developed soon after asphyxia and were followed by an asystolic heart rhythm with unmeasurable blood pressure. Cardiac arrest was defined as a mean femoral arterial pressure of less than 10 mmHg. In our prior study, the 72 h survival rate was 50% after 9.5 min asphyxia-induced cardiac arrest. We followed the protocol and applied in the current study [16]. After 9.5 min of asphyxia, epinephrine (0.01 mg/100 g) was administered through a venous line and chest compression was delivered by the index finger at a rate of 200 beats per minute by the same investigator for all animals. Chest compressions were adjusted to provide a uniform rate seen on monitoring and a target aortic diastolic pressure of > 20 mmHg during resuscitation. ROSC was usually achieved within 3 min, and animals were excluded if ROSC could not be obtained within 6 min. This was a randomized animal study to investigate the effects of OM treatment (n = 20 in each group, total n = 40). OM was continuously intravenously infused after ROSC at 0.25 mg/kg/h for 4 h in the experimental group. Normal saline was administered at an equivalent volume per hour for 4 h in the control group. Body temperature was maintained at 36.5°C during treatment (Fig 1).

### Survival study

Catheters and endotracheal tubes were removed from all animals after 4 h of invasive monitoring. Animals received an intraperitoneal injection of 1 mL 0.9% saline within 1 h after extubation and were returned to their cages. The health status of all rats was carefully monitored every 6 h. Survival status was recorded, and mortality was confirmed by loss of spontaneous respiratory movement and loss of heart beat for more than 2 min. The survival rates between the different groups were compared. If the animals reached humane endpoint criteria including body weight less than 20% compared to baseline or persistent seizure more than 10 minutes, we performed euthanasia immediately. All animals died before meeting criteria for euthanasia because animals usually died suddenly in the cardiac arrest and resuscitation experiment.

### Evaluation of neurological outcome

Recovery of neurological function was evaluated by neurological functioning scores for rats at the 6, 24, 48, and 72 h after cardiac arrest [14]. Neurological functioning scores included level of consciousness, corneal reflex, respirations, righting reflex, coordination, and movement/activity. The scores of each item were summed up. Neurological functioning scores (0–12) of 11 or 12 were defined as favorable neurological outcomes. Assessment of neurological function was independently performed by two investigators who were blinded to the treatment group. Any discrepancies were resolved by an independent assessment by a third investigator, and the scores chosen by the majority were accepted. After the evaluation of survival status and neurological outcomes were completed at 72 h after cardiac arrest, all animals were anesthetized with pentobarbital sodium (50 mg/kg) intraperitoneally for histological study.

### Histological examinations of the brain

To evaluate cerebral damage, brain were harvested at 72 h after cardiac arrest with rats anesthetized by pentobarbital sodium (50 mg/kg) intraperitoneally. Morphological and histological results of the brains were examined by hematoxylin and eosin staining. Healthy neurons had oval nuclei with prominent nucleoli and a lack of eosinophilic cytoplasm [17, 18]. The cornu amonis (CA1, CA2, CA3) in the hippocampus of each brain was selected for brain injury studies. Damaged neurons were counted in three independent, randomly selected microscopic fields of each hippocampal area. Histological examinations were performed by two independent pathologists who were blinded to the groups. If there were any discrepancies, an independent assessment was carried out by a third pathologist, and the majority opinion was chosen.

### Statistical analysis

Values of continuous variables are shown as mean ± standard deviation. Mixed linear models were used to compare the patterns and changes in hemodynamic parameters between the OM-treated and control groups. Survival curves were determined by the Kaplan-Meier method and compared using the log-rank test. Values of P<0.05 were considered statistically significant. Statistical analyses were performed using SPSS 21 software (SPSS Inc., Chicago, IL, USA).

## Results

### OM treatment improved the post-cardiac arrest cardiac function

**Fig 2:**
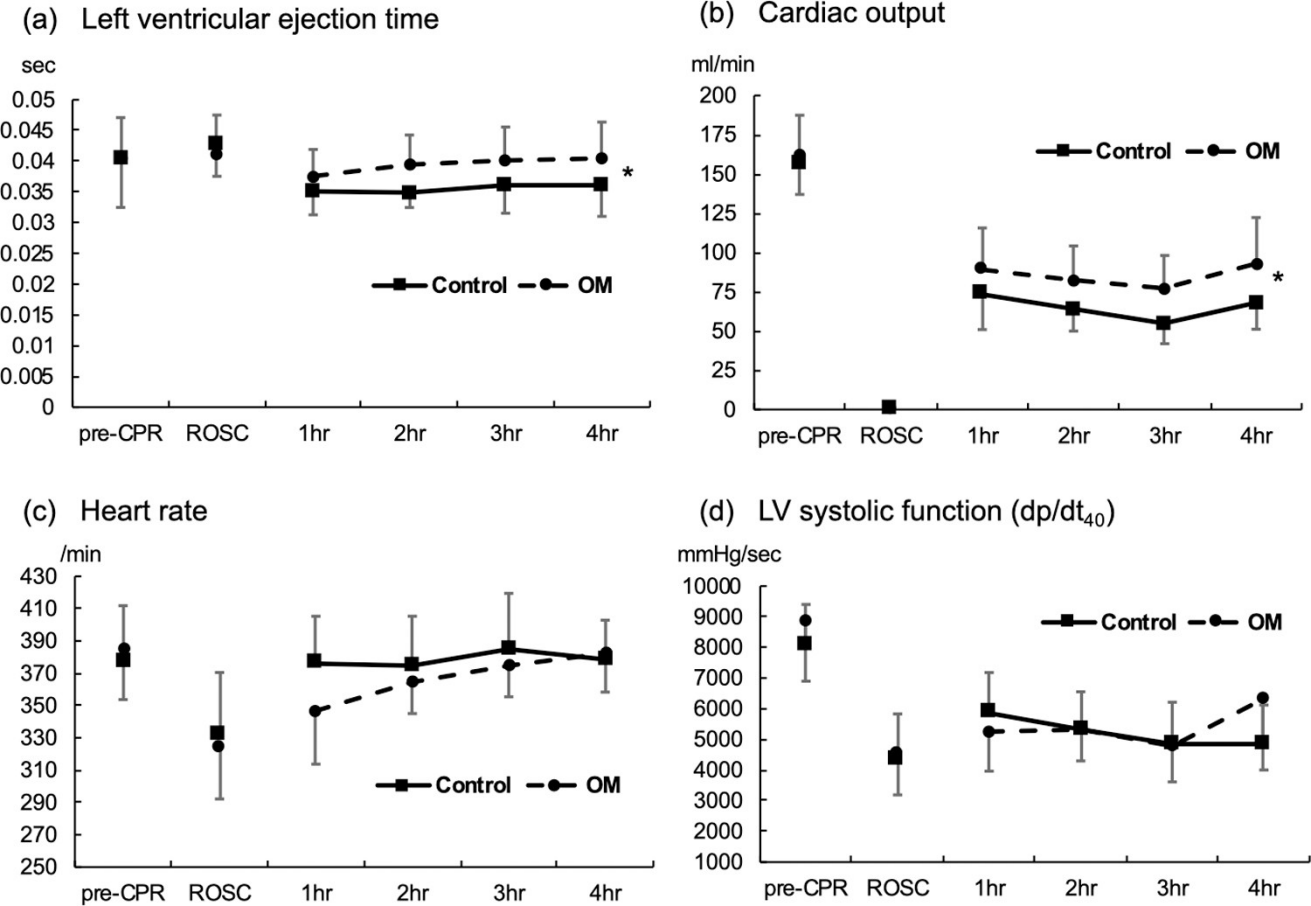
Hemodynamic parameters of control and OM groups following cardiac arrest and resuscitation. (a) Left ventricular ejection time. (b) Cardiac output. (c) Heart rate. (d) Left ventricular systolic function represented by dp/dt_40_. (n = 20 in each group, *: P<0.05 between two groups by mixed linear model analysis).

Hemodynamic parameters before inducing cardiac arrest and at ROSC were not significantly different between the two groups (Fig 2). Left ventricular ejection time was prolonged in the OM group compared to that in the control group from 1 h to 4 h after ROSC (Fig 2A; P = 0.009 by mixed linear model analysis). Cardiac output decreased after cardiac arrest and CPR and was undetectable immediately after ROSC. The recovery of cardiac output was better in the OM group at 1 h to 4 h post-resuscitation (Fig 2B; P = 0.038 by mixed linear model analysis). Heart rate was lower at first hour after ROSC in OM group. However, heart rate was not different between the two groups through the post-resuscitation period by mixed linear model analysis (Fig 2C). Left ventricular systolic function, represented by dp/dt_40_, showed no significant differences between the two groups after cardiac arrest and resuscitation (Fig 2D).

### Survival study

**Fig 3:**
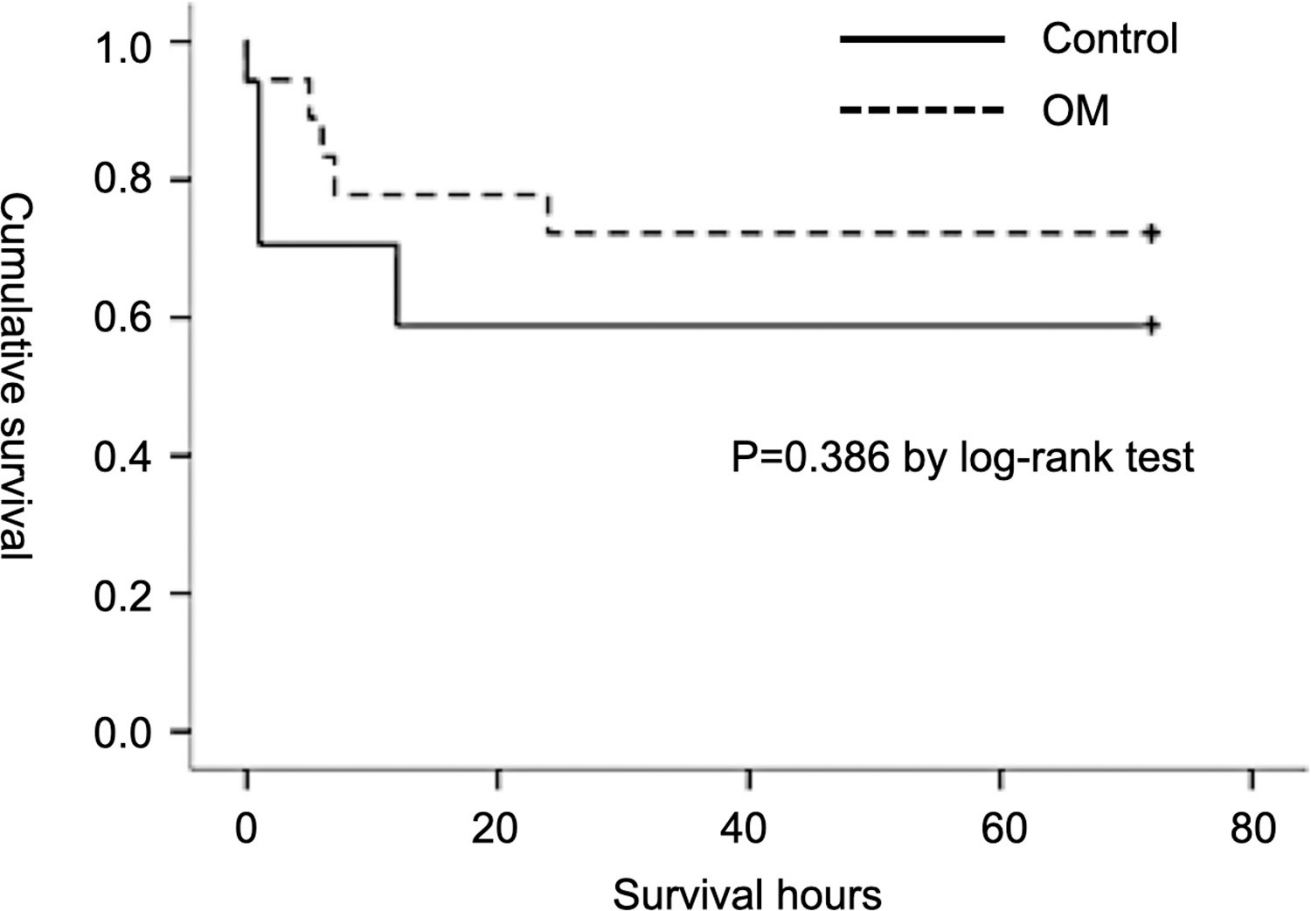
Kaplan-Meier survival curve of control and OM groups 72 h after cardiac arrest and resuscitation. (P = 0.386 by log-rank test).

The ROSC rate was 90% (18/20) in the OM group and 85% (17/20) in the control group (p = 0.633). Total mortality rate before ROSC was 12.5% (5/40). Survival status was monitored for 72 h. The survival rate of the OM group was 72.2% (13/18), and that of the control group was 58.8% (10/17). Kaplan-Meier survival curve analysis showed a higher survival rate in the OM group, but there were no significant differences between the two groups (Fig 3; p = 0.386 by log-rank test).

### OM treatment improved neurological outcome

**Fig 4:**
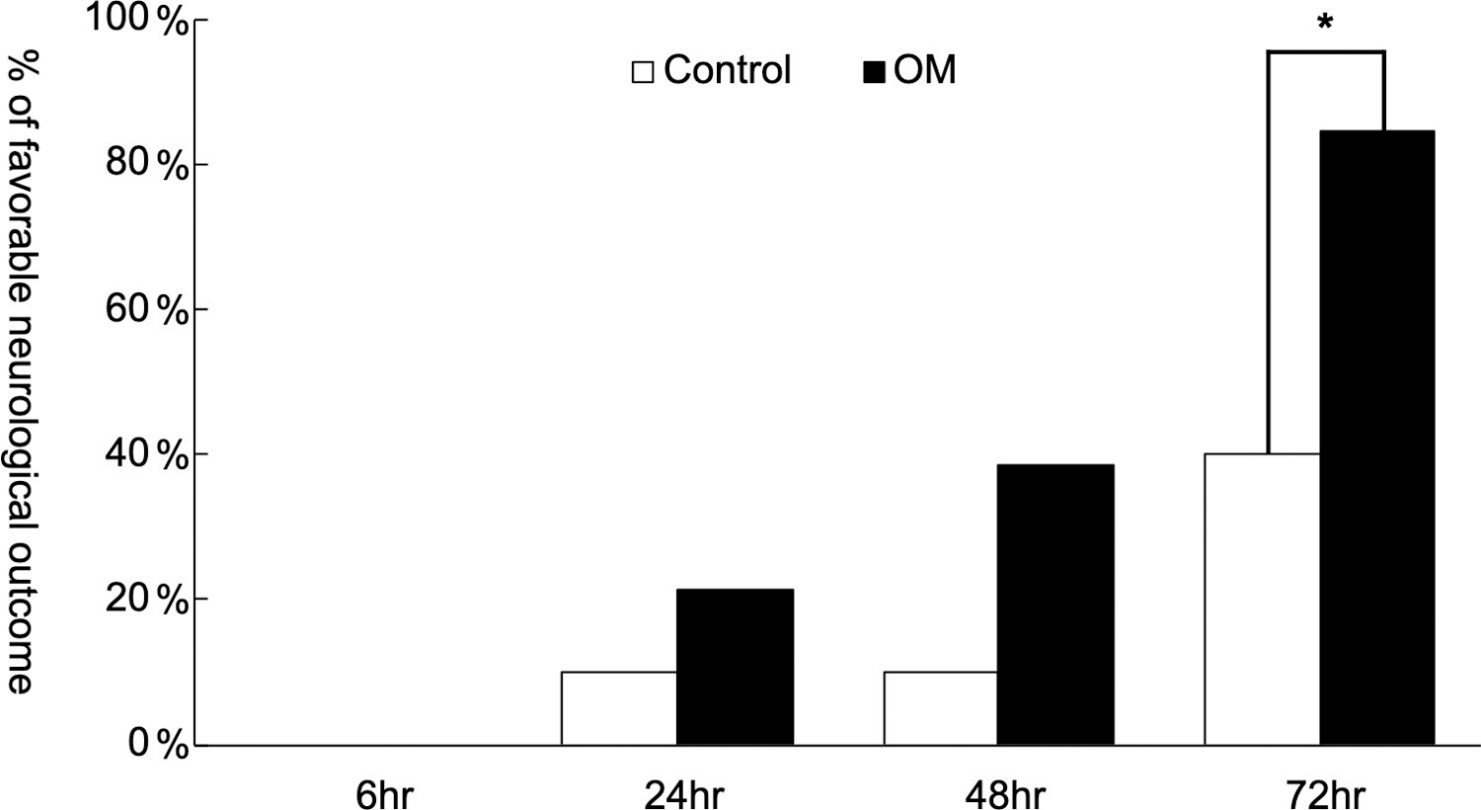
Neurological outcome of control and OM groups at 6, 24, 48 and 72 h after cardiac arrest and resuscitation. (*: P = 0.026).

The recovery of neurological function in surviving animals was evaluated using the neurological functioning score system. The OM group had a higher chance of having favorable neurological outcomes at 24, 48, and 72 h after cardiac arrest and resuscitation. There were significant differences between the OM group (11/13, 84.6%) and the control group (4/10, 40%) in having favorable neurological outcome at 72 h after cardiac arrest in the surviving animals (Fig 4; p = 0.026).

### OM treatment decreased neuron damage

**Fig 5:**
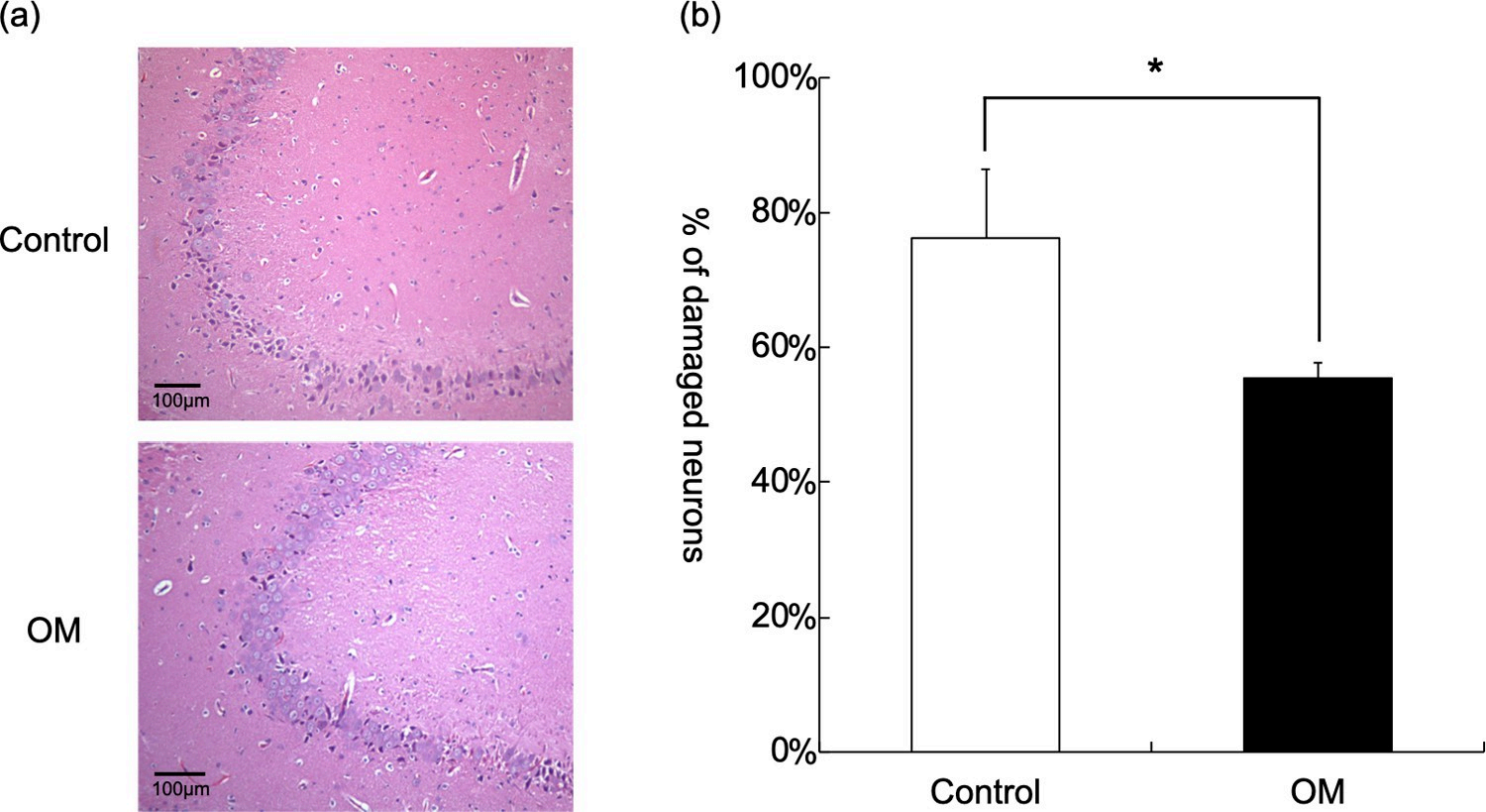
Histological examinations of the brain in control and OM groups. (a) Hematoxylin and eosin staining in brains 72 h after cardiac arrest. (b) Quantification of damaged neurons in each group. (*: P = 0.004).

In brain histological studies with hematoxylin and eosin staining, more severely damaged neurons had small-sized, darker, and condensed cytoplasm (Fig 5A). The OM group (55.5±2.3%) had a lower percentage of damaged neurons compared to that in the control group (76.2±10.2%) at 72 h after cardiac arrest (Fig 5B; p = 0.004).

## Discussion

Post-cardiac arrest myocardial dysfunction following cardiac arrest and resuscitation is one of the major causes of poor clinical outcomes and mortality. In the current study, we demonstrated that omecamtiv mecarbil (OM) treatment improved post-resuscitation cardiac output by prolonging left ventricular ejection time. However, heart rate and left ventricular systolic function were not affected by OM administration. OM treatment also reduced cerebral neuron damage and improved neurological outcome during the post-cardiac arrest period.

Several studies reported that nearly two-thirds of patients resuscitated from cardiac arrest had post-cardiac arrest myocardial dysfunction, even in the absence of prior cardiac disease [19]. Post-cardiac arrest myocardial dysfunction and profound shock contribute to multiple organ failure and mortality [3, 4, 20]. Inotropic support may be needed to optimize cardiac output, blood pressure, and systemic perfusion. Dobutamine can effectively ameliorate post-resuscitation systolic and diastolic dysfunction by stimulating beta-adrenergic receptors [21–23]. However, inotropic agents have several side effects, including a higher risk of arrhythmia, exacerbation of myocardial ischemia, and increased myocardial oxygen consumption and cardiomyocyte calcium transients [13, 23, 24]. OM is a selective cardiac myosin activator that augments the rate of ATP turnover, thus accelerating the transition rate from a weakly bound to a strongly bound actin-myosin complex. In contrast to inotropic agents, which augment cardiac contractility by increasing myocardial contraction rate and shortening systolic duration, OM increases cardiac function and stroke volume by extending the duration of ejection in the absence of changes in the heart rate [10, 12]. OM administration did not increase the overall myocardial oxygen demand or calcium transients [9, 10]. In this study, we demonstrated that OM treatment prolonged the left ventricular ejection time and thus improved post-resuscitation cardiac output without affecting heart rate or left ventricular systolic function.

In our study, the OM group had a higher survival rate, but there were no statistically significant differences between the two groups. The small number of experimental animals may have resulted in the lack of a statistical difference between groups. Studies have shown that deaths within the first 24 h after ROSC mostly result from cardiac disorders, while later deaths usually result from neurological injury [19, 25, 26]. Laurent et al. reported that resuscitated patients with a persistent low cardiac index at 24 h were more likely to experience early death due to multi-organ failure [3]. The separation of survival curves of the control and OM groups within 24 h was observed in our study. OM treatment may have certain survival benefits due to improvement of post-resuscitation cardiac function. Future research is needed to explore the relationship between OM treatment and survival outcome in patients resuscitated from cardiac arrest.

Cerebral injury occurs after cardiac arrest and leads to poor neurological outcomes. Decreased cerebral blood flow during the post-cardiac arrest period contributes to decreased cerebral oxygen supply, which causes hypoxic-ischemic brain injury [4, 27–29]. The low cardiac output caused by myocardial dysfunction may exacerbate hypoperfusion of vital organs and hence decrease brain perfusion in cardiac arrest survivors. Chang et al. demonstrated that patients successfully resuscitated from cardiac arrest who had a left ventricular ejection fraction (LVEF) greater than 40% were related to better neurological recovery [30]. Current guidelines recommend that resuscitated patients who have ST segment elevation on ECG or highly suspected acute myocardial infarction should undergo immediate coronary angiography with subsequent percutaneous coronary intervention (PCI) [21]. Several studies have reported that early PCI after cardiac arrest is associated with improved survival and neurological function [31–33]. The better survival and neurological outcomes in revascularized patients may be attributed to reduced myocardial ischemia and thus amelioration of cardiac function. In the current study, we demonstrated that resuscitated animals with OM treatment had a higher chance of having favorable neurological outcomes. In histological examinations at 72 h after cardiac arrest, a lower percentage of cerebral neuron damage was observed in the OM group.

Multiple interactive processes lead to myocardial dysfunction in cardiac arrest survivors. The pathophysiology of post-cardiac arrest myocardial dysfunction involves cardiovascular ischemia/reperfusion injury, excessive levels of inflammatory cytokine activation, and catecholamine-induced cardiovascular toxicity [19, 34]. Dobutamine can augment myocardial contractility by stimulating beta-adrenergic receptors and thus ameliorating post-resuscitation systolic and diastolic dysfunction [21–23]. Milrinone, a selective phosphodiesterase III (PDE III) inhibitor, can elevate intracellular cAMP in cardiomyocytes and increase contractility. In a ventricular fibrillation-induced cardiac arrest animal study, milrinone attenuates left ventricular dysfunction after resuscitation without exacerbating myocardial oxygen demand [35]. Levosimendan is a calcium sensitizer that increases myocardial contractility by sensitizing cardiac troponin C to the action of calcium and improves cardiac function in cardiac arrest [16]. The coadministration of levosimendan, epinephrine, and atenolol was beneficial for post-resuscitation myocardial function and 48-h survival in an animal model [36, 37]. The present study demonstrated that OM administration can improve post-resuscitation cardiac output and neurological outcome. Combination pharmacotherapy for the treatment of post-cardiac arrest myocardial dysfunction may have potential value to improve overall outcomes in post-resuscitation patients. More studies are needed to evaluate which combination of drugs can achieve the best therapeutic effect.

### Limitations

There are a few limitations in the current study. First, we used healthy experimental animals without comorbidities for the cardiac arrest model. In clinical practice, OM treatment may have different influences on cardiac arrest survivors with pre-existing medical conditions. More studies using a diseased model may be needed before translation to clinical use. Second, the average age of the experimental animals was 12 weeks old in our study. A recent study demonstrated that aging plays an important role in the function of cardiac muscle. Aging alters acetylation status in cardiac muscle which may contribute to muscle deterioration [38]. Further studies to evaluate the outcomes of the OM treatment in elder animals should be taken into consideration. Third, cardiac arrest was induced by the asphyxia model rather than the ventricular fibrillation (VF) model. Several studies reported that asphyxial cardiac arrest produces worse morphologic brain damage and neurologic injury, whereas VF cardiac arrest produces worse cardiovascular injury based on higher peak troponin and lower left ventricular ejection fraction [39–41]. Different mechanisms of cardiac arrest may have the potential to influence the treatment outcomes. Fourth, the dosage and duration of OM treatment was based on previous clinical studies on patients with acute heart failure. Further studies on efficacy and safety of OM treatment in cardiac arrest patients may be needed.

## Conclusions

In conclusion, OM treatment can improve post-resuscitation myocardial dysfunction by prolonging the left ventricular ejection time, reduce cerebral neuron damage, and improve neurological outcome in an animal model. These findings could be the basis for further pre-clinical studies to improve the outcomes in post-cardiac arrest care.

## Supporting information

S1 Table Neurological functioning scores in the study. Total possible score, 0 to 12.
(PDF)
