# Supplementary material for: Omecamtiv mecarbil treatment improves post-resuscitation cardiac function and neurological outcome in a rat model
Source: PLoS One. 2022 Feb 17;17(2):e0264165. doi: 10.1371/journal.pone.0264165 (PMC8853579; doi:10.1371/journal.pone.0264165)
Supplement: S1 Table — Total possible score, 0 to 12. (PDF) [file pone.0264165.s001.pdf]

S1 Table. Neurological functioning scores in the study. Total score, 0 to 12.

Score	Level of consciousness	Corneal reflex	Respirations	Righting reflex	Coordination	Movement/ activity
0	No reaction to pinching of tail	No blinking	Irregular breathing pattern	No turning attempts	No movement	No spontaneous movement
1	Poor response to tail pinch	Sluggish blinking	Decreased breathing frequency, normal pattern	Sluggish turning	Moderate ataxia	Sluggish movement
2	Normal response to tail pinch	Normal blinking	Normal breathing frequency and pattern	Turns over quickly and spontaneously	Normal coordination	Normal movement
